# The epidemiology of scorpion stings in tropical areas of Kermanshah province, Iran, during 2008 and 2009

**DOI:** 10.1186/s40409-015-0045-4

**Published:** 2015-11-05

**Authors:** Alireza Khatony, Alireza Abdi, Tahereh Fatahpour, Farhad Towhidi

**Affiliations:** School of Nursing and Midwifery, Kermanshah University of Medical Sciences, Kermanshah, Iran; Abolfazl Hospital of Ghasre-shirin, Kermanshah University of Medical Sciences, Kermanshah, Iran; Imam Reza Hospital, Kermanshah University of Medical Sciences, Kermanshah, Iran

**Keywords:** Scorpion stings, Epidemiology, Tropical climate, Kermanshah, Incidence

## Abstract

**Background:**

Scorpion stings are an acute health problem in tropical regions. Awareness of this problem is fundamental for establishing preventive interventions, thus prompting the present study to determine the scorpion-sting incidence in tropical areas of Kermanshah province during 2008 and 2009.

**Methods:**

In a retrospective study, all records related to scorpion sting patients from the health centers of tropical areas of Kermanshah were studied by a census and checklist. Data were analyzed by the software SPSS-16 using descriptive and inferential tests.

**Results:**

The incidence of scorpion stings was 334.37/100,000 inhabitants in 2008 and 339.07/100000 in 2009. Mean and standard deviation of age were 30.55 ± 16.99. Scorpion stings were more common in rural areas (59.6 %) and occurred more often in summer (52.9 %). Nearly 48 % of bites were to patients’ hands and 47.5 % of patients were injured between midnight and 6 a.m. While 92.9 % of patients had mild symptoms, scorpion antivenom was prescribed to 88.8 % of victims, 94.5 % of whom were discharged after outpatient treatment. The relationship between antivenom therapy and clinical symptoms was not significant.

**Conclusions:**

Due to the relatively high incidence of scorpion stings in tropical areas of Kermanshah, it is recommended that the inhabitants be educated through the mass media about how to prevent the stings and apply preliminary treatment.

## Background

Scorpion stings are a life-threatening emergency, and are considered one of the most important health challenges in tropical and sub-tropical regions [1–3]. There are no available accurate statistics on scorpion stings worldwide, but the literature indicates that all settings usually affected by this problem, as well as geographical characteristics and health facilities, affect outcomes, which are serious in some regions [4]. It is recognized that of the more than 1500 scorpion species in the world, few cause severe toxicity; the reports represent more than 1.23 million stings annually, of which approximately 3250 (0.27 %) cause death [1, 3–6].

Previous studies indicate a high prevalence of scorpion envenomation as well as related mortality in developing countries compared to developed nations, reflecting a lack of adequate health care facilities, low socioeconomic backgrounds, and inadequate authentic information about this affliction in poor regions [7–9]. In a related review study by Chippaux and Goyffon [6], seven areas were identified as more at risk, including Saharan Africa (north), Sahelian Africa, South Africa, the Near and Middle-East, southern India, Mexico and southern Latin America, and the region east of the Andes, where the total at risk population is 2.3 billion. In Middle Eastern countries there are almost 52 toxic scorpion species, most of which are found in Iran, due to its favorable geographic and climatic indicators [10]. More than 42,500 cases of scorpion stings per year in Iran have been reported, of which, despite appropriate treatments, nearly 20 individuals have died, while many were admitted to critical care units with irreversible cardiovascular and kidney disorders [10, 11].

In recent decades, although medical science has advanced, scorpion envenomation has been ignored for two main reasons, namely its unknown prevalence and a high number of low-income victims. Other factors that may increase its prevalence include poor managerial policy of resources and the cost of treatments, which contribute to higher mortality rates and related injuries [12]. In this aspect, in order to reduce the incidence of scorpion stings and its complications, the 4th International Conference on Snakebites and Scorpion Stings in Dakar, stressed the need to develop better recording and reporting systems, and optimize the gathering of data on scorpion injuries [13]. Chippaux [12] also stated that understanding the prevalence of scorpion stings and the rate of its complications in a local area is essential for designing preventive measures to reduce mortality and other burdens.

Due to special weather and geographical conditions, and their populations of more than 170,000, the cities of Ghasre-Shirin, Gilan-e Gharb and Sarpol-e Zahab, in tropical areas of Kermanshah province, are constantly faced with the problem of scorpion envenomation. Due to the lack of comprehensive information on this issue, the present study was conducted to determine the incidence of scorpion stings and related complications in tropical areas of Kermanshah during 2008 and 2009.

## Methods

**Fig. 1:**
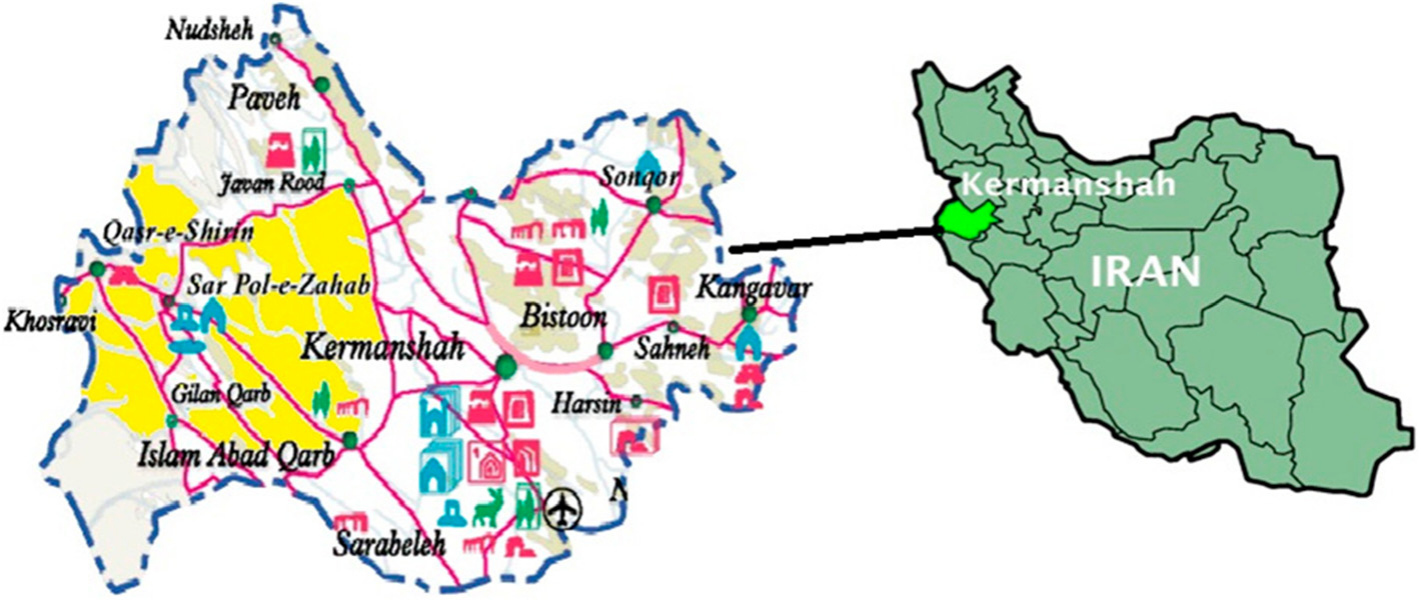
The location of the tropical areas of Kermanshah province in Iran, marked in yellow

The subjects of this descriptive-analytical retrospective study were all inhabitants of the tropical regions of Kermanshah during sampling. Kermanshah province, located in the west of Iran, presents various climates; Ghasre-Shirin, Gilan-e Gharb and Sarpol-e Zahab are tropical regions of the province (Fig. 1). The population for measuring the incidence of scorpion stings was estimated by referring to the Statistics Center of Iran [14], using the 2006 and 2011 censuses, which determined an annual growth rate of 1.179 % [15]. The sampling was done using the census method, by which all of the records of stings by scorpions during 2008 and 2009 were assessed.

The instrument was a researcher-tailored checklist made in accordance with the patients’ records and the special scorpion sting form. The checklist included demographic characteristics (age, sex, living location, admitted date, and job) and questions about where the victim was bitten, injured organs, time of the bite (12 p.m–6 a.m., 6 a.m.–12 a.m., 12 a.m.–6 p.m. and 6 p.m.–12 p.m.), referral hours after biting, scorpion color, clinical symptoms, patient status after initial treatment, and use of scorpion antivenom.

The therapy is based on polyvalent antivenom manufactured by Razi Vaccine and Serum Research Institute, which neutralizes the venom of six dangerous scorpion species including *Androctonus crassicauda*, *Buthotus saulcyi*, *Buthotus schack*, *Odontobothus doriae*, *Mesobuthus eupeus*, and *Hemiscorpius lepturus*. The antivenom is injected as intramuscular or slow intravenous infusion, usually 1 to 2 vials according to body weight, previous health, and age. The clinical symptoms were placed into three categories including: mild (no symptoms, localized signs, mild pain, malaise), moderate (severe malaise, drowsiness, nausea, vomiting, tachycardia, diaphoresis, and increased blood pressure) and severe symptoms (muscle spasm, dyspnea, acute pulmonary edema, cardiac disorders, hemoglobin disorders, and hematuria).

Data were collected by referring to the health centers of the research settings, after obtaining permission from the research deputy of Kermanshah University of Medical Sciences (KUMS) and health network officials in the cities of Ghasre-Shirin, Gilan-e Gharb and Sarpol-e Zahab. For this purpose all records related to scorpion stings during 2008 and 2009 were assessed, and then the questionnaires were completed. Data were entered into the software Statistical Package for Social Sciences, 16th version (SPSS v.16.0; SPSS Inc., USA) and analyzed by descriptive (frequency percent, mean and standard deviation) and inferential (chi-square for qualitative variables, one-way ANOVA for comparing the mean age in three clinical symptoms categories) statistics; the incidence was calculated by a special formula [16] and the confidence interval (CI) was estimated at 95 %. The significance level for all tests was 0.05.

### Ethical considerations

This study is the result of the research project n. 90,253, which was approved by the research committee of Kermanshah University of Medical Sciences (KUMS). Permission was also obtained from the health networks of Ghasre-Shirin, Gilan-e Gharb and Sarpol-e Zahab cities.

## Results

The total number of scorpion stings recorded during 2008 and 2009 was 1151, 568 (49.3 %) of which occurred in 2008 and the rest in 2009 (Table 1). The overall incidence, which was calculated from the scorpion sting frequency (1151 cases) and the population rate (about 170,000), was estimated at between 334.37 and 339.7 per 100,000 inhabitants in 2008 and 2009 respectively (Table 2); 697 (60.06 %) of patients were male, 59.6 % lived in a rural area, whereas the mean and standard deviation (SD) of age were 30.55 ± 16.94 years. Of this group, 33.2 % were in the 20–29 age group, 8.2 % (*n* = 94) were less than 10 years old and 7.9 % (*n* = 92) were older than 60 years. The chi-square test showed no significant difference between age and the symptoms (Table 3).

**Table 1:** The frequency of scorpion stings in the tropical area of Kermanshah

Year	2008	2009	Total
City	Frequency (%)	Frequency (%)	Frequency (%)
Ghasre-Shirin	170 (29.9)	156 (26.8)	326 (28.3)
Gilan-e Gharb	133 (23.4)	156 (26.8)	289 (25.1)
Sarpol-e Zahab	265 (46.7)	271 (46.5)	536 (46.6)
Total	568 (100)	583 (100)	1151 (100)

**Table 2:** The prevalence of scorpion stings per 100,000 people and its confidence interval in the tropical area of Kermanshah

Year	2008			2009		
City	Population	Prevalence	Confidence interval (95 % CI)	Population	Prevalence	Confidence interval (95 % CI)
Ghasre-Shirin	24,915	682.32	578.09–786.55	25,216	618.66	519.89–717.42
Gilan-e Gharb	61,376	216.70	179.09–254.30	62,117	251.14	210.97–291.30
Sarpol-e Zahab	83,598	316.69	279.48–355.60	84,607	320.30	281.64–358.97
Total	169,889	334.37	306.59–362.08	171,940	339.07	311.30–366.84

**Table 3:** Difference among age groups as to symptoms

Symptoms	Mild	Moderate	Severe	Total
Age groups (year)	frequency (%)	frequency (%)	frequency (%)	frequency (%)
<10	89 (8.3)	2 (9.5)	3 (4.9)	94 (8.2)
10–19	168 (15.7)	3 (14.3)	9 (14.8)	180 (15.6)
20–29	355 (33.2)	5 (23.8)	22 (36.1)	382 (33.2)
30–39	165 (15.4)	6 (28.6)	8 (13.1)	179 (15.6)
40–49	116 (10.9)	2 (9.5)	10 (16.4)	128 (11.1)
50–50	92 (8.6)	2 (9.5)	2 (3.3)	96 (8.3)
>60	84 (7.9)	1 (4.8)	7 (11.5)	92 (7.9)
Total	1069 (100)	21 (100)	61 (100)	1151 (100)
Statistical test	*χ* ^2^ = 11.11 *p* = 0.677			

**Fig. 2:**
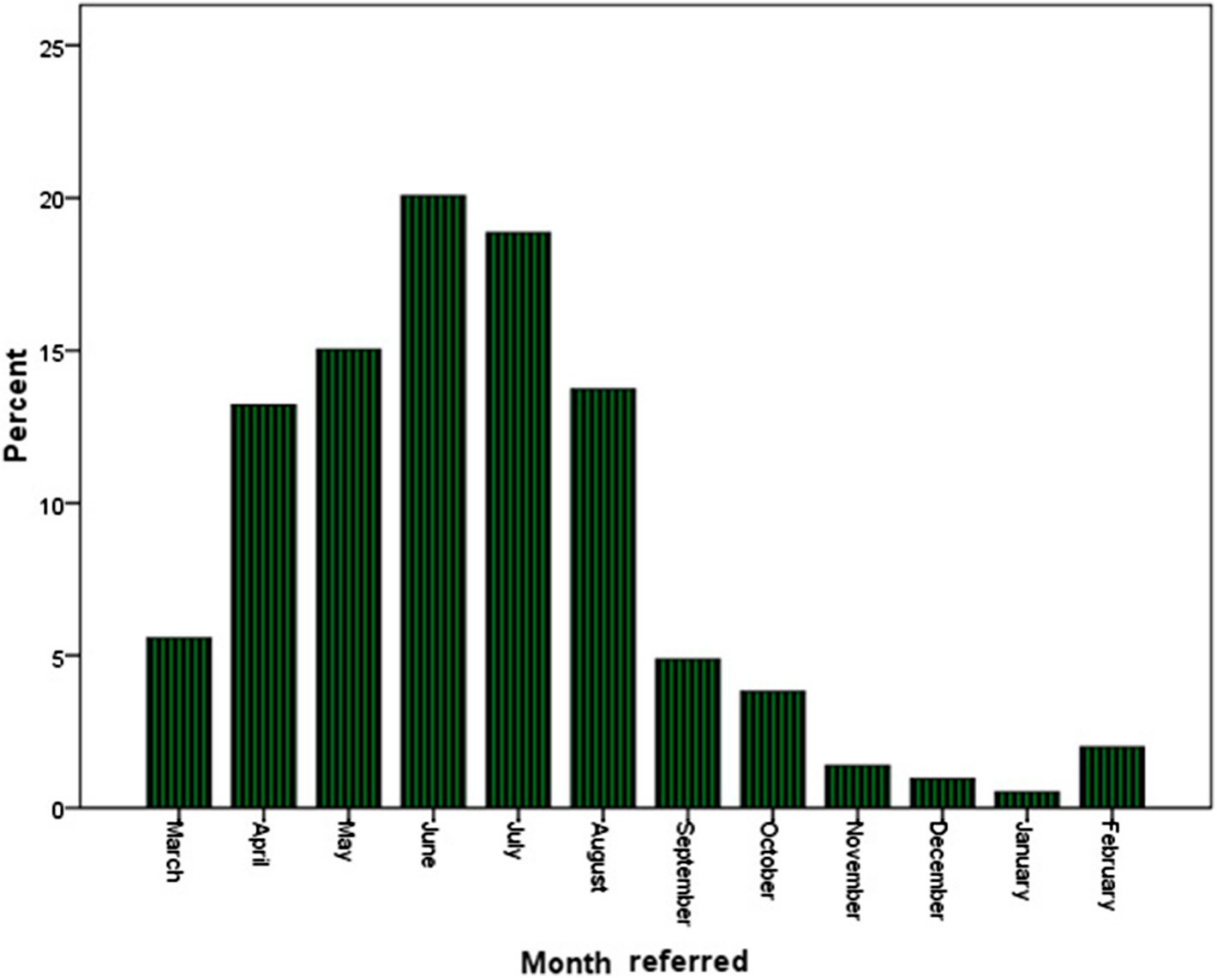
Frequency percent of scorpion stings based on the months of the year

As to occupation frequency, the housekeepers (28.3 %), students (17.2 %) and military members (14.2 %) ranked highest, with the results differing among the three cities (Table 4). About 53 % of scorpion stings happened in summer with most of them occurring in June (Fig. 2), with 58.2 % (664 cases) having occurred in roofed buildings and the rest in non-roofed buildings. In relation to color, 709 scorpions (61.6 %) were yellow, 347 (30.1 %) were black and in 95 cases (8.3 %) of unknown color. The most commonly injured organs were hands (48.4 %, 557 cases), feet (33.8 %, 389 cases), trunk (12.7 %, 146 cases), and head and neck (5.1 %, 58 cases). As to sting time, 47.5 % (547) happened from midnight to 6 a.m., 247 (21.5 %) cases from 6 p.m. to midnight and 195 cases (14 %) between noon and 6 p.m. Of all patients, 89.2 % (1027 cases) were admitted to health centers from midnight to 6 a.m.

**Table 4:** Frequency distribution of scorpion stings according to occupation in tropical areas of Kermanshah

City	Ghasre-Shirin	Sarpol-e Zahab	Gilan-e Gharb	Total	Statistical test
Occupation	Frequency (%)	Frequency (%)	Frequency (%)	Frequency (%)	
Employee	15 (4.6)	15 (2.8)	16 (5.5)	46 (4.0)	*χ* ^2^ = 174.90
Worker	13 (4.0)	23 (4.3)	23 (8.0)	59 (5.1)	*p* < 0.001
Farmer	19 (5.8)	64 (11.9)	35 (12.1)	118 (10.3)	Df = 14
Military	111 (34.2)	36 (6.7)	16 (5.5)	163 (14.2)	
Unemployed	27 (8.3)	33 (6.2)	45 (15.6)	105 (9.1)	
Housekeeper	61 (18.8)	183 (34.1)	82 (28.4)	326 (28.3)	
Self-employment	30 (9.2)	78 (14.6)	27 (9.3)	135 (11.7)	
Student	49 (15.1)	104 (19.4)	45 (15.6)	198 (17.2)

The symptoms of 92.9 % (1062 cases) were mild, 1.8 % (21 cases) moderate, and 5.3 % (61 cases) had severe clinical manifestations. Corticosteroid and sedative medications were prescribed for all patients, while scorpion antivenom was used in 88.8 % (1019 patients). There was no significant relationship between clinical symptoms and scorpion antivenom by the chi-square test (*χ*^2^ = 5.43, *p* = 0.066). Most patients (94.5 %, *n* = 1088) were treated as outpatients, while 5.5 % (63 cases) had been dispatched to other better-equipped centers. There was no difference in clinical symptoms in terms of living location, injured organ, biting time, scorpion color, sex, and season. During the research period, no deaths related to scorpion envenomation had been reported.

## Discussion

The incidence of scorpion stings was estimated at 334.37 and 339.07 individuals per 100,000 in 2008 and 2009, respectively, in the current study. These rates are higher than other cities in Iran such as Bushehr, Hormozgan, Sistan and Baluchestan, and Ilam, where incidences are 127, 153.9, 136.1 and 123 per 100,000, respectively. The mean incidence in Iran was measured as 59/100,000 annually, being lowest (zero) in Mazandaran (north of Iran) and highest in Khuzestan (south of Iran) (541/100,000) [17]. A study by Bouree et al. [18] in Mexico found a scorpion sting incidence of 584/100,000 in 1994 and 2043/100,000 in 2003, which are higher than our study. Research results indicate high rates of scorpion envenomation in Brazil [19] and Algeria [20], but lower ones in Singapore [21]. Investigations also demonstrated that all regions in the world other than Antarctica are vulnerable to scorpions, but the problem is more serious in tropical and subtropical countries [17]. Chowell et al. [7] argue that in areas with rainfall lower than 30 mm/year, and temperatures below 16 °C, scorpion envenomation decreases. Other researchers also believe that in areas where the people do not adhere to safety precautions and where substandard housing and dense trees predominate, the prevalence of stings will be higher [5]. Hosseininasab et al. [22] argue that houses with large cracks, the presence of firewood and wood shavings near human residences, and the behavior of sleeping in open spaces are predisposing factors of scorpion stings. Its high incidence in the tropical area of Kermanshah may be caused by some factors such as warmer weather conditions, the existence of substandard old houses, lack of adherence to safety precautions such as wearing adequate shoes and socks, living in unsprayed areas, resting in the outdoors, and insufficient supply of information to inhabitants about measures to prevent scorpion envenomation.

Most of the patients evaluated in the present study had mild symptoms, and there were no cases of death. These results are in accordance with those of Al Asmari et al. [23] in Saudi Arabia, and Sagheb et al. [11] in Shiraz, Iran; but Bouree et al. [18] determined a fatality frequency of more than 1000 individuals, while Shahbazzadeh et al. [24] reported three deaths in Khuzestan, Iran. The research indicated that the most common causes of death after a scorpion sting are disturbances in respiratory and cardiovascular systems, as well as hematological disorders such as disseminated intravascular coagulation (DIC), which differ according to scorpion species, proximity to medical centers, specific organs injured, and when treatment is started [25, 26]. Of the 45,500 people affected by scorpion bites in Iran, nearby 20 individuals have died annually, most of whom were bitten by one of ten scorpion types among the Buthidae species [10]. Victims of these species, if not treated in a timely manner, will die, because the scorpion protein toxin disturbs sodium and potassium channels in cells [27]. Due to a scarcity of studies, the common scorpion types in this study settings are unknown, but in the neighboring provinces such as Ilam seven scorpion species were more prevalent: *Buthotus saulcyi*, *Androctonus crassicauda*, *Scorpio maurus*, *Mesobuthus eupeus*, *Hemiscorpius lepturus*, *Compsobuthus matthiesseni* and *Odontobuthus doriae*, whose lethality varies [28]. It appears that the survival of victims in the present study can be explained by early referral of victims to medical centers, low toxicity of the scorpions in these areas, the dispatching of critically ill patients to other centers and inability to follow their treatment processes, which demanded more surveys.

We found out that scorpion antivenom was prescribed for more than two thirds of patients, and the relationship between its use and clinical symptoms was not significant. Similar results were obtained by Al Asmari et al. [23] and Abdolaeifard et al. [29]. In Khaderi’s study [30] in Khuzestan, Iran, patients envenomed by scorpions were treated only with corticosteroid and sedative medications without using antivenom. In this aspect Mortazavi-Moghadam [31] stressed only symptomatic therapy, maintaining that, due to numerous side effects, antivenom is unnecessary. According to the World Health Organization (WHO), the indicators for using scorpion antivenom are not well known and there are some controversies [32]. Some researchers also suggested revising the recommendations for use of scorpion antivenom, but in general most experts have emphasized the use of this drug for patients with severe symptoms who are under ten years old [30]. Bernstein [33] also stated that the use antivenom should be individualized by weighing the risk of administrating an immune serum with the level of available supportive care, the cost of supportive care, and the cost of obtaining or importing the drug. In Iran, most antivenoms are produced by Razi Vaccine and Serum Research Institute [34]. Scorpion antivenoms are administered according to the immunization guide approved by the Ministry of Health and Medical Education of Iran based on patients conditions [35]. Although antivenom therapy is undertaken according to the manufacturers’ instructions, these differences are justified [36]. It appears that in our study, because of poor follow-up systems, decisions about prescribing antivenom are also affected by factors such as physicians’ fear of legal issues related to the lack of scorpion antivenom, and the mental stability of patients after its administration. Thus, the absence of a relationship between use of antivenom and clinical symptoms is probably due to the high proportion of mild cases, as antivenom neutralizes circulating toxins [36, 37]. Thus, detailed information about the evolution of the patients submitted to serum therapy is essential to establish strategies of preventive measures for an at-risk population, which demanded more attention.

In the present study, the scorpion stings were more frequent in the 20–29 year age group, which corresponded to the findings of Hellal et al. [20] in France and Al Asmari et al. [23] in Saudi Arabia. However, Talebian and Dorodgar [38] and Hosseininasab et al. [22] reported that the age groups more affected were under 10 and 10–19 years, respectively. In the present study, it seems that the high frequency in the 20–29 year age group may be related to young people working in outdoor locations such as farms and gardens, and the existence of several military barracks, whose members are mostly young.

We observed that most scorpion stings occurred in summer and June, which is in accordance with many previous studies, because scorpions are more active in warm weather and summer [22, 38, 39]. In the study by Talebian and Dorodgar [38], resting in open spaces and non-compliance with the safety precautions have been mentioned as increasing the likelihood of stings in the summer. In the present study, besides the abovementioned findings including more people working outdoors in summer harvesting crops and living in substandard military barracks and houses, the increasing numbers of scorpion sting cases in summer is to be expected.

In the current work, more males than females were affected by scorpion stings, which is similar to the studies by Hosseininasab et al. [22] and Talebian and Dorodgar [38]. However, researchers from the USA [40] and Turkey [39] found that women were more affected. The difference among studies regarding gender may be related to demographic characteristics and active forces in these regions. For example, in the tropical area of Kermanshah, most of the people who work in outdoor and military settings are male; therefore, it is expected that more of them will be stung by scorpions.

The present results showed no significant difference among the age groups in terms of clinical symptoms. However, in other studies, children and elderly people were more vulnerable, and also presented more serious complications [11, 41, 42]. Some researchers also indicated that because in children and elderly the body mass is low and the blood–brain barrier (BBB) is more permeable to scorpion toxin, severe symptoms and mortality are expected to be high [42–44]. We believe that the negligible difference in symptoms in children and the elderly compared to other groups may be related to families and healthcare workers paying more attention to quick transference of patients to health centers and careful follow-up of the treatment status.

The present findings indicate that more than 57 % of scorpion stings occurred in roofed buildings, whereas in the Brazilian study by Amorim et al. [45], 90 % of sting were also received under covered space, but this rate was found to be 42 % by Hosseininasab et al. [22] in Kerman, Iran. It appears that in the geographical conditions of the tropical area of Kermanshah where most of the houses are of substandard construction, some of which have been temporarily and unsafely built on farms and military barracks, the occupants while sleeping are unaware of their surroundings, causing an increase in stings in roofed buildings.

In our study, the most common sting period was from midnight to morning (6 a.m.), which is similar to other studies in Saudi Arab [23]; Kashan, Iran [38]; and Khuzestan, Iran [24]. The high prevalence of stings may be related to high activity of scorpions at night, because in general, during the daytime, scorpions are in safe places such as cracks of walls, under rocks, between the leaves and debris around buildings, even in shoes and slippers, and they go out to hunt at night [22]. In addition, factors such as lack of visibility of scorpions in the dark and individuals being asleep also are important.

According to our observations, scorpion stings were more prevalent in rural areas than urban environments, which is in accordance with the findings of Bosnak et al. in Turkey [5]. However, Pipelzadeh et al. [25] in Ahwaz, Iran, reported that stings occurred more frequently in urban areas. In rural regions, most of the people work on farmlands and outside of the home, creating predisposing factors envenomations [39]. Additionally, the number of scorpions is higher on farmlands and in animal barns, in rural regions [38]. The researcher believes that these factors in rural areas with large houses and many trees provide an environment conducive to scorpion activity.

Following up the status of patients who were dispatched to other centers was impossible in the present work, so this was accepted as a non-control constraint. In the region of the study, some who may have suffered a scorpion sting were not referred to a health center, and thus did not have their experience recorded. There were also some limitations in relation to the items in the records such as the identification of the scorpion types and their lethality, uncertainty about the details of stings and admission times. In this aspect, it is recommended that some qualitative research with purposive sampling be undertaken as well as qualitative and quantitative studies on how health care workers cared for the envenomed patients and identified the scorpion types in the tropical areas of Kermanshah. Furthermore, it is suggested that some measures to dedicate resources such as equipment and qualified workforces to the health sectors as well as modification of the items recorded should be done.

## Conclusion

The results of our study indicate a high incidence of scorpion stings in the tropical areas of Kermanshah. Therefore, it is suggested that residents should be educated about safety precautions in relation to this problem. Additionally, the renovation of dilapidated urban and rural buildings, the equipping of health centers with essential facilities, and attending to the living environments of the military forces is recommended. Besides basic measures of environmental management for preventing scorpion bites, it is necessary to update the clinical management to the patients due to misuse of antivenom therapy.

## Abbreviations

KUMS: Kermanshah University of Medical Sciences
ANOVA: Analysis of variance
CI: Confidence interval
SD: Standard deviation
